# PLASMA NEUROFILAMENT LIGHT CHAIN IS ASSOCIATED WITH PHYSICAL FRAILTY IN OLDER ADULTS WITHOUT DEMENTIA

**DOI:** 10.1093/geroni/igad104.1657

**Published:** 2023-12-21

**Authors:** Junxin LI, Jessica Gill, Jing Huang, Daniel Waszczuk, Sarah L Szanton

**Affiliations:** Johns Hopkins University, Baltimore, Maryland, United States; Johns Hopkins University, Baltimore, Maryland, United States; Johns Hopkins University, Baltimore, Maryland, United States; Johns Hopkins University, Baltimore, Maryland, United States; Johns Hopkins University, Baltimore, Maryland, United States

## Abstract

Evidence suggests a link between brain biomarkers of neurodegeneration and physical function and frailty. However, the association of neurodegenerative biomarkers in the plasma with physical frailty in older adults remains unelucidated, as current evidence is limited and inconsistent. We tested the association between plasma neurofilament light chain (NFL), a plasma neurodegeneration biomarker, and physical frailty in a sample of community-dwelling older adults. We used baseline demographics, frailty, and plasma NFL data collected from 97 participants in a randomized controlled trial in community-dwelling, sedentary older adults without dementia [Montreal Cognitive Assessment (MoCA)>17]. Physical frailty was assessed using a composite measure of weakness, slow walking speed, unintentional weight loss, exhaustion, and low physical activity. Participants’ frailty levels were categorized into robustness, pre-intermediate frailty, and frailty. Multiple linear regression and ordinal logistic regression models adjusting for age, sex, race, education, and comorbidities were conducted to test the associations of NFL with frailty and levels of frailty. The sample was 70.0 ± 6.0 years old, with 80% being females, 72% having intact cognition, and 28% having mild cognitive impairment (MCI). In the adjusted models, higher plasma NFL was associated with higher frailty scores (β=0.16, 95%CI [Confidence Interval] = [0.06,0.26]) and a higher likelihood of being in a higher level of frailty group (odds ratio=1.28, 95% CI =1.02, 1.61). In summary, plasma NFL was associated with greater frailty in our sample of non-demented older adults. Plasma NFL may be a sensitive and promising neurodegenerative biomarker for physical frailty in older adults without dementia.

